# Lactylation: a metabolic–epigenetic driver in atherosclerosis pathogenesis and therapeutic targeting

**DOI:** 10.3389/fcvm.2026.1790479

**Published:** 2026-06-26

**Authors:** Wenbo Lv, Linxi Xie, Jintao Tao, Qingqi Xu, Wenfeng Hu, Hao Xie, Pin Lu, Ying Xu, Liang Huang

**Affiliations:** 1 Research Laboratory of Translational Medicine, Hengyang Medical School, University of South China, Hengyang, Hunan, China; 2 Departments of Clinical Medicine, Hengyang Medical School, University of South China, Hengyang, Hunan, China

**Keywords:** atherosclerosis, epigenetic regulation, lactate metabolism, lactylation, vascular inflammation

## Abstract

Atherosclerosis (AS), a disease of large and medium-sized arteries, is a common cause of cardiovascular morbidity and mortality. Lactylation is a recently identified post-translational modification involving the addition of lactate-derived lactyl groups to lysine residues on proteins. Studies suggest that lactylation plays a vital role in AS development by regulating several key pathological processes. These include inflammation, epithelial–mesenchymal transition, angiogenesis, vascular smooth muscle cell senescence and transdifferentiation, and metabolic dysregulation associated with atherosclerosis. This review summarizes the molecular mechanisms through which lactylation contributes to AS initiation and progression, providing a clearer understanding of the underlying pathophysiological processes. Further elucidation of lactylation may provide novel mechanistic insights into AS development and identify lactylation-targeted interventions as promising strategies to slow AS progression and reduce cardiovascular events.

## 1 Introduction

Lactate, the main byproduct of anaerobic glycolysis, accumulates in the cytosol when pyruvate is reduced by lactate dehydrogenase. This tetrameric oxidoreductase exists as six distinct isoenzymes assembled from the three gene products LDHA, LDHB, and LDHC, whose variable composition determines kinetic preference for either pyruvate reduction or lactate oxidation (1). Previous studies have shown that cancer cells generate energy through glycolysis under both aerobic and anaerobic conditions via the Warburg effect, leading to lactate accumulation (2). Research indicates that monocarboxylate transporters (MCTs) facilitate the proton-dependent transport of lactic acid across the plasma membrane and within the cytoplasm and intracellular compartments. This process enables lactic acid to function as both an energy substrate and a signaling molecule that regulates various physiological functions (3, 4).

Lactylation, a recently identified post-translational modification, plays a crucial role in modulating the function, stability, subcellular localization, and interactions of various proteins, encompassing both histones and non-histone proteins (5). Research has demonstrated that lactate is converted into lactyl-CoA, which is then transferred to lysine residues on target proteins through the action of lactyltransferases, thereby affecting relevant signaling pathways and regulating biological processes and disease progression. Previous studies demonstrated that the histone acetyltransferase p300 may function as a lactyltransferase, mediating the lactylation modification process of histones H3 and H4 (6). In a related study, specific siRNA was used to knock down p300 and cyclic adenosine monophosphate response element-binding protein (CREB)-binding protein (CBP). Following the reduction of p300/ CBP activity and expression, HMGB1 lactylation levels were observed to decrease significantly, supporting the role of p300/CBP as lactyltransferases. After reducing the activity and expression of p300 and CBP, it was observed that the lactylation level of HMGB1 significantly decreased. These findings support the potential role of p300/CBP as lactyltransferases (7). In addition, delactylases participate in lactylation regulation, acting in opposition to lactyltransferases. Research has indicated that class I histone deacetylases, specifically histone deacetylase (HDAC) 3, HDAC4, and HDAC5, may exhibit delactylase activity, suggesting their potential role as “erasers” in the regulation of lactylation by removing lactyl groups (8). In another study, an *in vitro* screening system identified nuclear HDAC1-3 as the most efficient delactylase for lactylated lysine. Moreover, class III histone deacetylases, such as sirtuin (SIRT) 1–3, exhibit significant delactylase activity, indicating their potential as effective “erasers” in lactylation modification (9).

**Figure 1:**
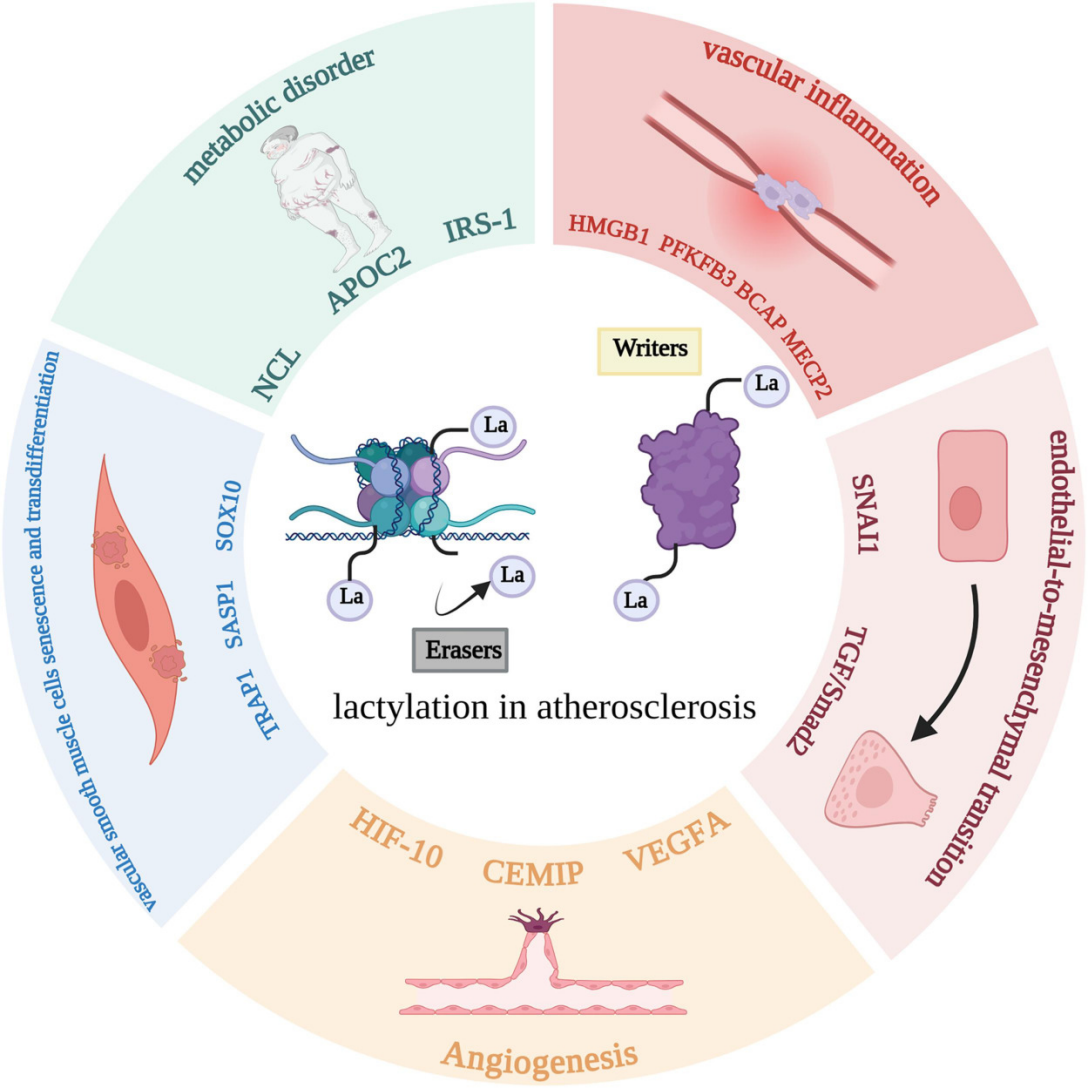
Schematic overview of lactylation in atherosclerosis, illustrating its roles in vascular inflammation, endothelial-to-mesenchymal transition, angiogenesis, vascular smooth muscle cell phenotypic switching, and metabolic dysregulation. Created in BioRender. lv, W. (2026) https://BioRender.com/s307yvu.

Atherosclerosis (AS), a chronic arterial disease, is the primary cause of coronary heart disease, stroke, and other cardiovascular conditions (10). Lactylation is involved in the initiation and progression of atherosclerosis (11, 12). Current theories propose that the onset and progression of AS are primarily driven by vascular inflammation, endothelial–mesenchymal transition, angiogenesis, vascular smooth muscle cell (VSMC) senescence and transdifferentiation, and metabolic disorders (13–17). Understanding the underlying molecular mechanisms is essential for prevention and management of AS. While the general molecular mechanisms and biological functions of protein lactylation have been well summarized in recent reviews (18, 19), a systematic, detailed discussion of its specific roles in the key pathological processes of AS remains lacking. This review examines the molecular mechanisms of lactylation in AS, highlighting its contributions in vascular inflammation, endothelial-to-mesenchymal transition (EndMT), angiogenesis, vascular smooth muscle cell senescence and transdifferentiation, and metabolic dysregulation associated with atherosclerosis. Importantly, the effects of lactylation across these processes are not independent; instead, they are regulated by a context-dependent framework that links metabolism, epigenetic regulation, and cell-type-specific responses (Figure 1).

## 2 Lactylation in the vascular inflammation

AS has been identified as a progressive chronic inflammatory disease of the vessel wall. Currently recognized risk factors for atherosclerosis include hypercholesterolemia, hypertension, hyperhomocysteinemia, diabetes, smoking, and obesity (20). Chronic exposure to risk factors can impair the endothelial barrier's function and morphology, leading to the secretion of chemokines that promote the infiltration of macrophages and other inflammatory cells, thereby triggering vascular inflammatory cascades and accelerating AS progression (13, 21). In this section, we highlight glycolysis-coupled lactylation mechanisms when their main pathological consequence is amplification of vascular inflammatory signaling.

HMGB1 is a multifunctional protein widely expressed in eukaryotic cells. HMGB1 functions intracellularly as a nuclear protein, while in the extracellular space, it primarily acts as an inflammatory mediator. It transmits relevant cellular signals through pattern recognition receptors, such as the receptor for advanced glycation end products (RAGE) and Toll-like receptors (TLRs), leading to the release of inflammatory cytokines and mediating the inflammatory response (22). Several molecules, including RAGE, SIRT1, and TLR4, are involved in the transcriptional control of HMGB1 and modulate its role in inflammation and immune responses by influencing its post-translational modifications and dynamic intracellular–extracellular translocation (23).

Evidence indicates that macrophages acquire extracellular lactate via MCTs. Imported lactate subsequently promotes HMGB1 lactylation through the p300/CBP complex. This lactylated form of HMGB1 is released into the extracellular milieu via exosomal pathways. This lactylated HMGB1 impairs endothelial integrity and increases vascular permeability. It reduces VE-cadherin and claudin-5 expression while increasing ICAM-1 levels. These changes accelerate AS progression (7).

Heat shock protein A12A (HSPA12A), an atypical member of the HSP70 family, has been shown to inhibit macrophage chemotaxis in mice. Overexpression of HSPA12A in hepatocytes could reduce serum HMGB1 levels and suppress HMGB1 lactylation and secretion. Mechanistically, HSPA12A inhibits HMGB1 lactylation by targeting glycolytic flux, thereby exerting a distinct regulatory role in the inflammatory cascade of AS. These observations collectively imply that HSPA12A is a novel regulatory factor capable of inhibiting macrophage chemotaxis and the subsequent inflammatory cascade by preventing glycolysis-mediated HMGB1 lactylation, thus reducing the risk of AS (24). Furthermore, it has been demonstrated that silencing lactate dehydrogenase A can reduce the accumulation of histone lactylation in the promoter regions of the HMGB1 gene. LDHA silencing also attenuates the cellular expression of IL-18 and IL-1β (25). Notably, silencing LDHA, a critical metabolic enzyme, may affect lactylation at the promoter regions of other genes. Thus, further studies are necessary to explore the potential anti-inflammatory mechanism of LDHA. Collectively, these findings suggest that inhibiting histone lactylation at the HMGB1 promoter and reducing HMGB1-related lactylation may suppress vascular inflammation, highlighting HMGB1 as a potential therapeutic focus in AS.

**Figure 2:**
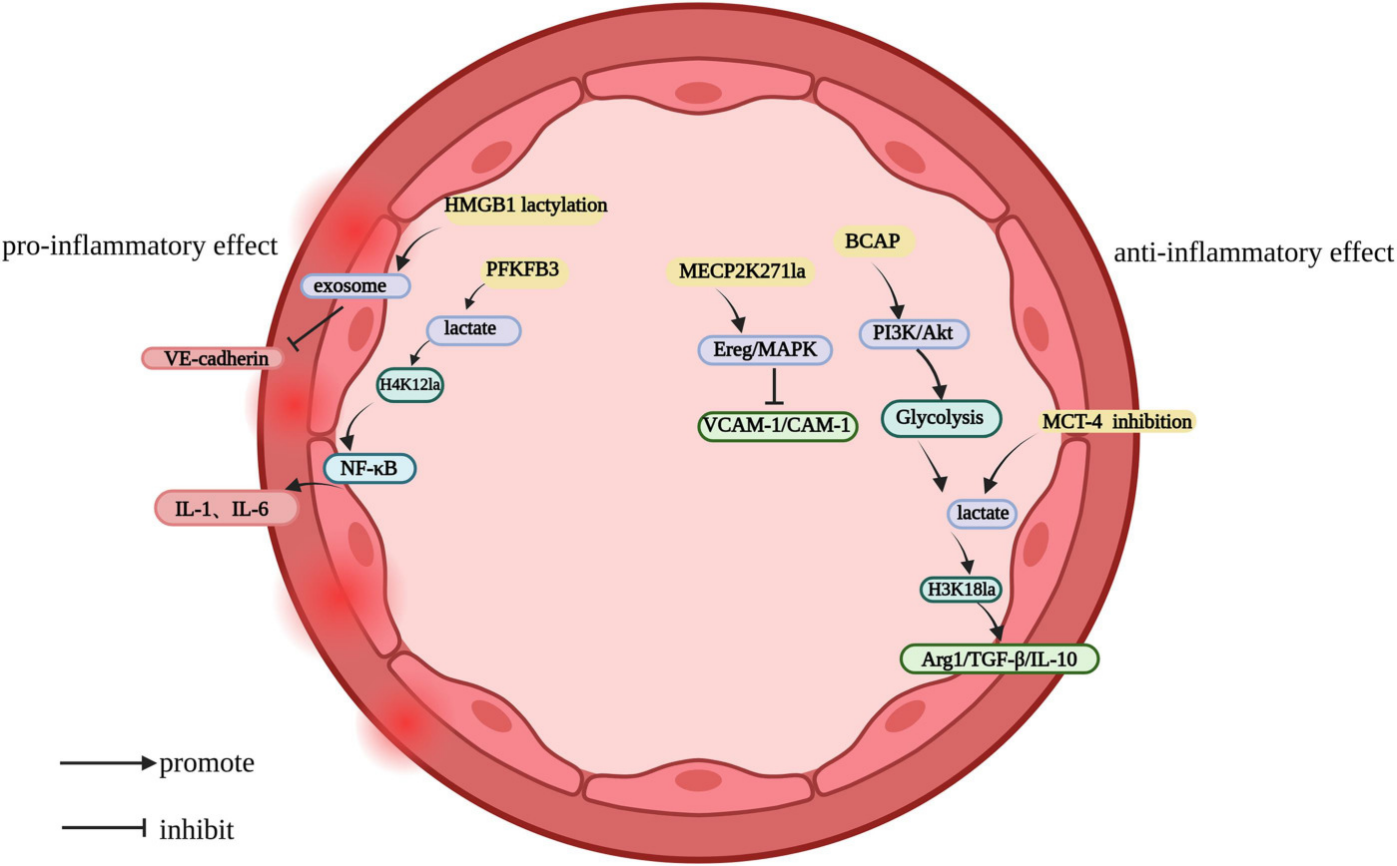
Dual roles of lactylation in vascular inflammation, illustrating both pro-inflammatory and anti-inflammatory mechanisms mediated by histone and non-histone lactylation. Created in BioRender. Lv, W. (2026) https://BioRender.com/drpzxau.

Studies indicate that pathogen-associated molecular patterns (PAMPs) and downstream inflammatory cytokines activate NF-*κ*B signaling, which not only drives transcription of IL-6 and IL-8 but is also further amplified by lactylation, thereby perpetuating and amplifying the inflammatory cascade central to atherosclerotic advancement (26). It has been reported that histone H3K18 lactylation (H3K18la) accumulates at the promoter of Ras homolog family member A (RhoA), which enhances RhoA/ Rho-associated coiled-coil containing protein kinase (ROCK)/Ezrin signaling and activates NF-*κ*B, ultimately mediating the inflammatory response. This provides evidence that lactylation contributes to the onset of inflammation (27). The 6-phosphofructo-2-kinase/fructose-2, 6-bisphosphatase 3 (PFKFB3) is a key enzyme controlling one of the most critical steps of glycolysis. Upregulated glycolytic activity drives lactate production, which simultaneously increases histone lactylation, particularly at histone H4 lysine 12 lactylation (H4K12la). Research indicates that H4K12la displays preferential accumulation at the promoter regions of NF-*κ*B target genes, thereby facilitating NF-*κ*B transcription and driving inflammatory reactions. PFKFB3 indirectly activates this pathway by enhancing glycolysis and increasing lactate production. Elevated lactate promotes H4K12la accumulation and upregulates IKK*β* and p65 expression. This metabolic–epigenetic circuit may amplify vascular inflammation and accelerate AS. Consistently, the PFKFB3 inhibitor 3PO lowers H4K12la, blunts NF-*κ*B activation, and reduces both inflammatory cytokine release and collagen deposition. Within atherosclerotic plaques, pharmacological PFKFB3 blockade thus alleviates inflammation mainly by suppressing glycolysis, limiting lactate accumulation, and consequently decreasing histone H4K12 lactylation (28) (Figure 2). Because the dominant output of this pathway is inflammatory amplification rather than metabolic homeostasis, it is discussed here under vascular inflammation.

Macrophages play a crucial role in both inflammation and atherosclerosis, undergoing dynamic phenotypic transitions. They are broadly characterized by two functionally distinct states: the M1 phenotype, which exhibits pro-inflammatory properties, and the M2 phenotype, which exerts anti-inflammatory effects and supports tissue repair and inflammation resolution (29). The metabolic profiles of macrophages are strongly associated with their phenotypes. M1 macrophages exhibit a metabolic profile dominated by aerobic glycolysis for energy generation, whereas M2 macrophages predominantly obtain their energy through fatty acid oxidation and oxidative phosphorylation (30). Lactic acid and lactylation play pivotal roles in controlling macrophage metabolism and suppressing macrophage activation and proliferation. Research has shown that lactylation of chromatin histones enhances gene transcription within chromatin and upregulates the expression of homeostatic genes such as arginase 1 (Arg1), leading to a shift in macrophages from a pro-inflammatory to an anti-inflammatory phenotype (6). These findings suggest that targeting the regulation of Arg1 expression through lactylation modification could represent a promising approach for treating vascular inflammation. Metabolic reprogramming driven by LDHA attenuates ROS and induces M2 macrophage polarization, alleviating the inflammatory response while promoting fibrotic repair during the resolution phase of inflammation (31). Emerging evidence indicates that plaque rupture and the ensuing coronary thrombosis precipitate myocardial infarction (MI), during which heightened glycolytic flux and impaired MCT1-mediated lactate export raise H3K18la levels. This, in turn, modulates the anti-inflammatory response of monocyte-derived macrophages, mitigating post-MI inflammation (32, 33). In addition, MCT4 (monocarboxylate transporter 4), which is robustly expressed by macrophages residing in atherosclerotic plaques, primarily mediates lactate efflux. Inhibition of MCT4 results in intracellular lactate accumulation. The buildup of lactate functions as a substrate for p300-mediated histone H3K18 lactylation, subsequently inducing anti-inflammatory gene expression (e.g., IL-10, TGF-*β*) and promoting the transition of macrophages toward a reparative M2 phenotype. This shift alleviates inflammation, improves metabolic reprogramming, and ultimately inhibits the progression of AS (34). Furthermore, B-cell adaptor for phosphoinositide 3-kinase (BCAP) plays a critical role in connecting TLR signaling with aerobic glycolysis by activating the PI3K–Akt–mTOR signaling axis. Upon activation by TLR ligands, such as lipopolysaccharide or pathogens, BCAP mediates the activation of the PI3K/Akt pathway. Akt, in turn, phosphorylates and inhibits downstream targets, including forkhead box O 1 (FOXO1) and glycogen synthase kinase 3 beta (GSK3*β*), limiting the excessive expression of NF-*κ*B-driven pro-inflammatory factors, such as IL-12. Simultaneously, Akt promotes the abundance of rate-limiting glycolytic enzymes, such as hexokinase (HK) 2 and LDHA, potentiating aerobic glycolysis and driving marked lactate production. Elevated intracellular lactate acts as a substrate for histone lactylation, a post-translational modification that directly influences gene expression. Histone lactylation promotes the interaction of chromatin with the promoter regions of repair-related genes, such as Arg1 and IL-10, thereby promoting their transcription. BCAP-driven histone lactylation facilitates macrophage polarization from an inflammatory to a pro-reparative phenotype. In contrast, macrophages deficient in BCAP exhibit impaired glycolysis and reduced lactate production, leading to markedly reduced histone lactylation levels, hindered repair gene expression, and delayed resolution of inflammation (35). By integrating TLR signaling with metabolic and epigenetic regulatory networks, BCAP emerges as a pivotal molecule in the inflammation-to-repair transition of macrophages. Modulating the BCAP/PI3K pathway or histone lactylation status constitutes an innovative therapeutic approach for AS.

Methyl-CpG-binding protein 2 (MECP2), an X-linked gene (chromosome Xq28), produces a nuclear factor that avidly binds methylated CpG islands in promoter regions, acting as a bidirectional transcriptional regulator and chromatin architecture modulator (36, 37). Research indicates that MECP2 functions as an essential epigenetic controller for macrophage responses to external stimuli and stress (38). Interestingly, recent findings have shown that exercise-induced MeCP2 K271la directly interacts with H3K36me3. This interaction suppresses SETD2-mediated H3K36 trimethylation at the RUNX1 promoter. As a result, RUNX1 transcription is repressed. These changes reduce vascular inflammation and improve plaque stability (39). Another study revealed that exercise-mediated endothelial MECP2 K271 lactylation (MECP2 k271la) downregulates the abundance of vascular cell adhesion molecule 1 (VCAM-1), intercellular adhesion molecule 1 (ICAM-1), monocyte chemoattractant protein 1 (MCP-1), IL-1β, and IL-6 through the activation of the Epiregulin/MAPK signaling pathway. In addition, MeCP2 K271la upregulates endothelial nitric oxide synthase levels in mouse aortic tissues, effectively suppressing vascular inflammation and reducing the progression of AS (12). These findings suggest that MeCP2 K271la represents a potential therapeutic target for modulating vascular inflammation and impeding the advancement of atherosclerosis.

Gegen Qinlian decoction was found to attenuate these pro-inflammatory effects by reducing lactate biosynthesis and the associated histone lactylation, thereby shifting macrophage polarization toward an anti-inflammatory M2 phenotype (40). This suggests that in this specific context, reducing H4K8/K12la drives an anti-inflammatory response. Conversely, lactylation can also orchestrate anti-inflammatory activities in other settings. For example, in Th17 cells, lactate induces an increase in H3K18la levels, which contributes to the conversion of inflammatory T lymphocytes into a regulatory T cell identity, thereby promoting inflammation resolution (41).

A mechanistic framework can reconcile the apparently contradictory roles of lactylation in vascular inflammation. Rather than representing opposing effects, pro-inflammatory and anti-inflammatory outcomes of lactylation reflect a context-dependent regulatory program determined by three interrelated dimensions: the temporal stage of inflammation, intracellular lactate availability, and cell-type-specific enzymatic and substrate selectivity. Lactylation acts at both chromatin and protein levels (42). It regulates transcriptional activation, transcription factor activity, and intercellular signaling. Together, these effects shape distinct inflammatory phenotypes (43).

First, lactylation exhibits stage-specific regulatory effects. During the early phase of inflammation, enhanced glycolysis drives rapid lactate production and promotes histone lactylation at promoters of NF-*κ*B target genes (28). This process strengthens inflammatory transcription through a feed-forward metabolic–epigenetic loop (44). In contrast, during the resolution phase, sustained lactate accumulation functions as an intrinsic timing signal that induces lactylation at reparative genes such as Arg1 (45, 46). This shift promotes macrophage polarization toward an anti-inflammatory phenotype and supports tissue repair (47). This temporal transition reflects metabolic reprogramming of macrophages from glycolysis toward oxidative metabolism, which changes the transcriptional output of lactylation. This concept is consistent with the lactate clock model that links early inflammation to later repair.

Second, lactate availability introduces a threshold-dependent regulatory effect (48). Lactate acts not only as a metabolic byproduct but also as a signaling molecule that determines the extent and genomic distribution of lactylation. When lactate is produced transiently during acute glycolysis, lactylation preferentially enhances inflammatory gene expression. When lactate accumulates persistently, widespread lactylation promotes the expression of anti-inflammatory and reparative genes, including IL-10 and TGF-*β* (46). This threshold-dependent effect may explain why lactylation exerts dual regulatory functions (49).

Third, cell-type-specific mechanisms further determine functional outcomes. In macrophages, lactylation mainly regulates chromatin accessibility and inflammatory gene programs through histone modification, thereby directly influencing inflammatory polarization and disease progression. For example, histone lactylation has been shown to promote macrophage M1 polarization and pro-inflammatory signaling through transcriptional activation of specific downstream targets, highlighting its role in driving inflammatory responses in vascular pathology (50). In contrast, in endothelial cells, lactylation contributes to vascular dysfunction through distinct mechanisms. Recent studies have demonstrated that glycolysis-driven lactate accumulation induces histone lactylation at key promoters involved in NF-*κ*B signaling, thereby enhancing endothelial inflammation and promoting early atherogenesis (51). Moreover, lactylation can also regulate endothelial structure and barrier integrity by modulating transcription factors and downstream targets involved in glycocalyx degradation and vascular permeability (52). These findings indicate that lactylation exerts cell-specific regulatory effects by targeting different molecular substrates and signaling pathways, ultimately shaping distinct pathological outcomes in immune and vascular compartments.

Taken together, lactylation functions as a metabolic–epigenetic rheostat rather than a simple pro- or anti-inflammatory signal. Its net effect is determined by when it occurs, how much lactate is available, and which cell type is involved. This integrated model explains why lactylation can enhance inflammation through glycolysis-driven histone modification while also promoting inflammation resolution through activation of reparative gene programs, thereby resolving the apparent paradox. Future studies should define quantitative lactate thresholds and characterize the spatial distribution of lactylation to improve mechanistic precision (53).

## 3 Lactylation in the endothelial-to-mesenchymal transition

**Figure 3:**
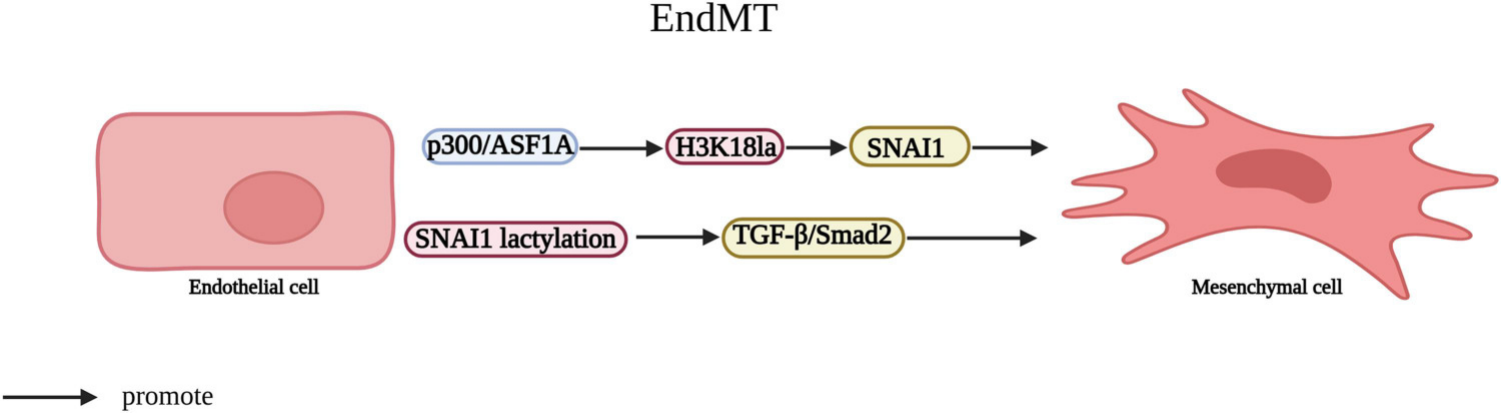
Mechanisms of lactylation-mediated endothelial-to-mesenchymal transition (EndMT), highlighting the roles of histone lactylation and key signaling pathways such as TGF-*β*/Smad. Created in BioRender. Lv, W. (2026) https://BioRender.com/crliqmx.

EndMT is a key process in the pathophysiology of AS through mediating endothelial disruption, intimal thickening, increased vascular stiffness, and plaque development (54). The origin of mesenchymal cells within atherosclerotic plaques has been extensively investigated. Advanced investigations of endothelial lineage dynamics have revealed that the endothelium serves as a major contributor to plaque-associated mesenchymal cells (14). EndMT enables endothelial lineage cells to acquire mesenchymal characteristics, marked by reciprocal downregulation of endothelial identity markers (VE-cadherin, CD31) and upregulation of mesenchymal signature proteins (*α*-SMA, N-cadherin, calponin) (55). Meanwhile, endothelial cell-specific functions, such as the capacity for angiogenesis, inhibition of thrombin formation, lectin-binding ability, and uptake of low-density lipoprotein cholesterol, are diminished or lost, while the characteristics of mesenchymal cells such as cell migration, invasion, contraction, and collagen secretion are enhanced (56). Notably, transforming growth factor-*β* (TGF-*β*) has been widely documented to critically regulate EndMT-driven fibrosis across diverse cardiovascular pathologies (57, 58). Emerging evidence suggests that lactylation contributes to the regulation of TGF-*β*-induced EndMT in vascular endothelial cells (59). Activation of TGF-*β* signaling leads to EndMT through the phosphorylation of Smad2/3 and the upregulation of transcription factors, such as snail family transcriptional repressor 1 (SNAI1) (60). Studies identified SNAI1 as a critical determinant of EndMT initiation and subsequent progression (61). *In vitro* lactate treatment has been shown to impair endothelial cell function and promote mesenchymal features under hypoxic conditions via activation of the TGF-*β*/Smad2 pathway. Mechanistically, lactate facilitates the interaction between CBP/p300 and SNAI1, leading to p300-mediated lactylation of histone H3 at lysine 18 (H3K18la) in a MCT-dependent manner. MCT inhibition effectively suppresses both lactate-induced EndMT and H3K18la enrichment (62). Targeting the lactylation sites of SNAI1 might offer an effective means to curb the progression of AS by modulating EndMT (Figure 3).

Studies demonstrate that oxidized low-density lipoprotein (ox-LDL) induces H3K18 lactylation in endothelial cells by promoting lactate production, which regulates SNAI1 expression and induces EndMT (63, 64). During the expression of SNAI1, the histone chaperone antisilencing function 1A (ASF1A) was identified as a key cofactor for P300 (65). It is essential for the precise regulation of H3K18la enrichment at the SNAI1 promoter, leading to the activation of SNAI1 transcription and promoting EndMT. Moreover, knockdown of ASF1A inhibits EndMT and ameliorates endothelial dysfunction (66). These findings indicate that the p300/ASF1A molecular complex mediates EndMT through H3K18la-dependent transcriptional activation of SNAI1, revealing a novel epigenetic mechanism and viable therapeutic objective for atherosclerosis therapy.

## 4 Lactylation in angiogenesis

Angiogenesis exerts dual effects in AS plaque progression. While neovascularization provides oxygen and nutrients to growing plaques, the primitive structure of intraplaque microvessels renders them susceptible to rupture, inciting plaque hemorrhage and culminating in destabilization (15). Emerging evidence implicates histone lactylation as a critical epigenetic modulator in this process.

Lactylation at histone H3 lysine 9 (H3K9la) has emerged as a hypoxia-sensitive epigenetic mark regulating angiogenic gene programs. Notably, in microvascular endothelial cells, stimulation of vascular endothelial growth factor (VEGF) specifically elevates H3K9la levels at the promoters of a set of pro-angiogenic genes, including nectin cell adhesion molecule 1 (NECTIN1), transforming growth factor beta receptor 2, and epidermal growth factor receptor, directly linking this modification to transcriptional activation of key factors in vessel growth (67). This modification establishes a feed-forward loop: VEGF-induced H3K9la promotes endothelial cell proliferation and tubulogenesis, while simultaneously suppressing histone deacetylase 2 (HDAC2), the primary eraser enzyme for histone lactylation (67). Pharmacological inhibition of HDAC2 exacerbated this loop, whereas HDAC2 overexpression attenuated H3K9 lactylation and pathological angiogenesis (67). Targeting this self-reinforcing H3K9la/HDAC2 axis may provide a potential strategy for stabilizing vulnerable plaques.

**Figure 4:**
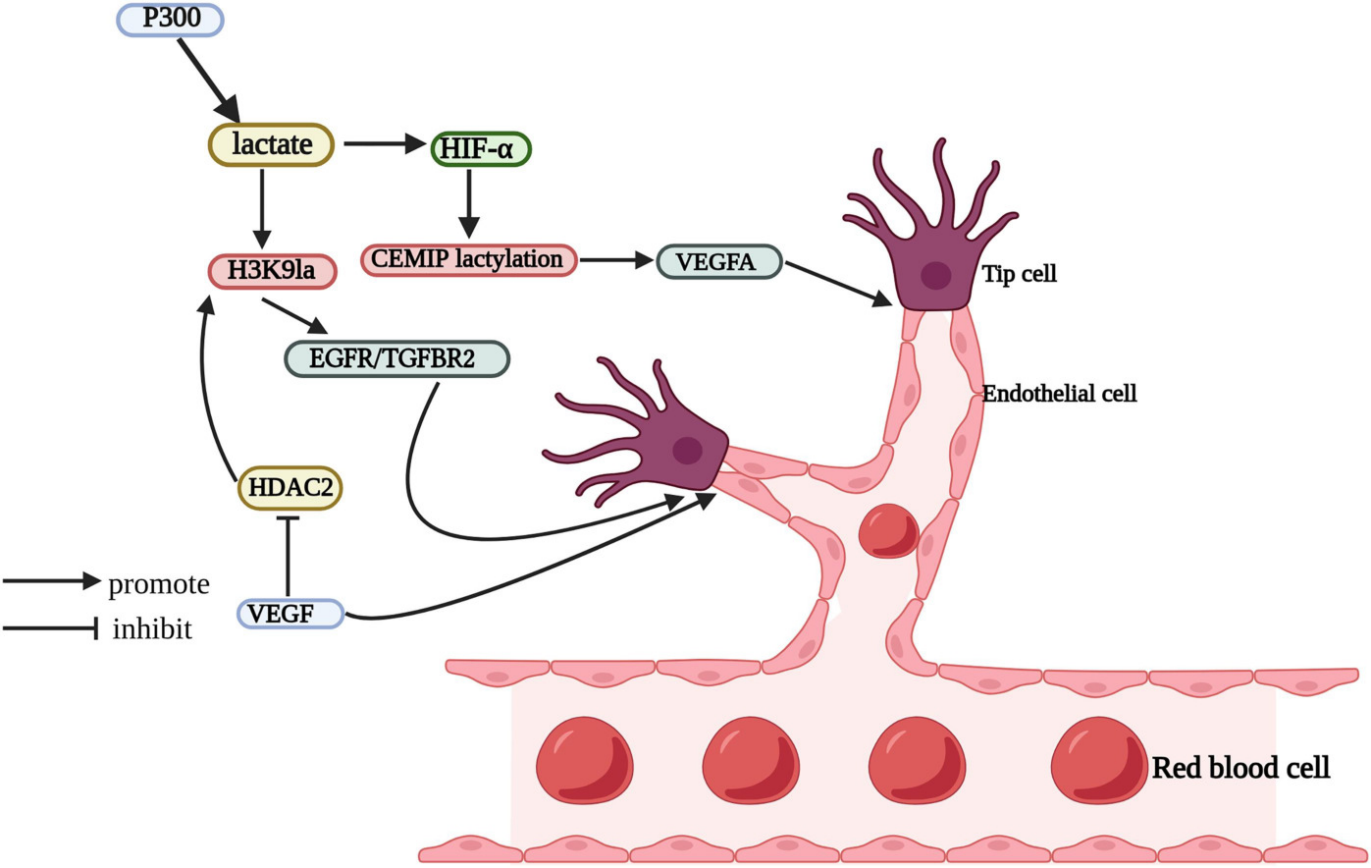
Lactylation-driven angiogenesis, illustrating how lactate-induced histone lactylation regulates pro-angiogenic factors such as VEGFA through HIF-1*α* signaling pathways. Created in BioRender. Lv, W. (2026) https://BioRender.com/w946gh2.

Hypoxia-inducible factor 1 (HIF-1) is a central mediator of hypoxia-induced angiogenesis. It functions as a heterodimer composed of the constitutively expressed subunit HIF-1β and the hypoxia-inducible subunit HIF-1*α* (68). HIF-1*α* regulates the expression of a variety of genes, such as endothelin-1, matrix metalloproteinases, and VEGF (69–71). The ox-LDL can stimulate angiogenesis by inducing HIF-1*α* expression in macrophages, thereby contributing to the instability of atherosclerotic plaques (72). Recent investigations have shown that cell migration-inducing protein (CEMIP, also known as KIAA1199), a hyaluronic acid-binding protein, is involved in pathways related to glycolysis, hypoxia, and angiogenesis. CEMIP promotes angiogenesis by modulating VEGFA expression (73, 74). MCT1, as a high-affinity lactate transporter, mediates the entry of lactate into cells, stabilizing HIF-1*α* protein levels under normoxic conditions. In particular, lactate internalized via MCT1 induces lactylation of HIF-1*α*, a covalent modification that augments HIF-1*α* stability and transcriptional function. Lactylated HIF-1*α* directly binds to a specific site on the CEMIP gene promoter region (−1,882 to −1,873) and upregulates CEMIP, catalyzing the degradation of high-molecular-weight hyaluronic acid into low-molecular-weight fragments. These fragments activate VEGFA signaling and suppress the antiangiogenic protein Semaphorin 3A (Sema3A). In addition, they promote the accumulation of VE-cadherin and phosphorylated Eph receptor A2 (EphA2), ultimately enhancing angiogenesis. Therefore, the MCT1-mediated lactate/HIF-1*α* lactylation/CEMIP transcriptional axis establishes a metabolic–epigenetic regulatory network under normoxic conditions, offering a novel molecular mechanism to explain the relationship between lactylation and angiogenesis (74) (Figure 4). Collectively, these findings reveal that HIF-1*α* lactylation drives intraplaque angiogenesis. Therefore, targeting this modification presents a potential therapeutic avenue for stabilizing atherosclerotic plaques.

## 5 Lactylation in the vascular smooth muscle cell senescence and transdifferentiation

VSMC senescence promotes atherosclerosis and plaque instability (16). Tumor necrosis factor receptor-associated protein 1 (TRAP1), an aging-related metabolic regulator, is prominently increased in atherosclerotic plaque regions. TRAP1 expression is elevated in the aortic tissues of both atherosclerotic patients and high-fat diet-fed ApoE deficiency mice, as well as in Ras-induced senescent human VSMCs, suggesting a close association with atherosclerosis. This upregulation is primarily driven by aberrant activation of Ras under pro-atherogenic conditions. In this context, TRAP1 enhances aerobic glycolysis, leading to increased lactate production. The accumulated lactate promotes lactylation of histone H4 lysine 12 (H4K12la) by downregulating the histone lysine delactylase HDAC3. H4K12la becomes enriched at promoters of senescence-associated secretory phenotype (SASP) genes, activating transcription of SASP genes and accelerating VSMC senescence (75). Thus, TRAP1-mediated metabolic reprogramming increases lactate-dependent H4K12la via HDAC3, facilitating SASP gene expression and unveiling a prospective therapeutic avenue for treating VSMC senescence in atherosclerosis.

**Figure 5:**
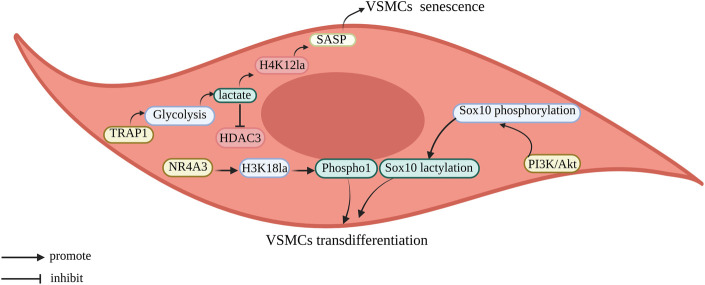
Roles of lactylation in vascular smooth muscle cell (VSMC) senescence and transdifferentiation, including metabolic reprogramming and signaling pathways involved in phenotypic switching. Created in BioRender. Lv, W. (2026) https://BioRender.com/oxbcwwx.

VSMCs undergo transdifferentiation into macrophage-like cells in response to chronic inflammatory injury, contributing to atherosclerosis complications (76, 77). PI3K/AKT signaling induces phosphorylation of SRY-box transcription factor 10 (Sox10) at serine 24 (S24). This phosphorylation is required for subsequent lactylation. This phosphorylation-dependent lactylation enhances Sox10 transcriptional activity. Activated Sox10 directly binds to the promoters of macrophage-associated genes, including C3, Cd74, and lysozyme. These transcriptional changes promote VSMC transdifferentiation into pro-inflammatory macrophage-like cells. In particular, lactylated Sox10 recruits chromatin modifiers to upregulate adhesion molecules (VCAM-1, MCP-1) and pyroptosis-related factors (gasdermin D caspase-1), which synergistically promote phagocytic behavior, immune cell recruitment, and vascular inflammation. Furthermore, regulator of G-protein signaling 5 (RGS5) suppresses this process by antagonizing PI3K/AKT-mediated Sox10 phosphorylation, illustrating the essential role of lactylated Sox10 in VSMC phenotype conversion and destabilization of atherosclerotic plaques (78) (Figure 5).

Vascular calcification is significantly associated with both increased all-cause mortality and destabilization of atherosclerotic plaques, thereby promoting plaque rupture, partly due to the transdifferentiation of VSMCs within the vessel wall (79). NR4A (nuclear receptor subfamily 4, group A), a transcription factor of the steroid–thyroid hormone–retinoid receptor superfamily, serves as an essential mediator of metabolic control and energy equilibrium (80). Phosphatase orphan 1 (Phospho1), a soluble phosphatase, facilitates calcium–phosphate mineral crystallization by hydrolyzing phosphocholine and phosphoethanolamine to release inorganic phosphate (Pi), a critical precursor for hydroxyapatite formation. Novel findings reveal that Phospho1 serves as a key driver in the osteogenic transdifferentiation of VSMCs, where its upregulation directly promotes medial vascular calcification. Preclinical validation has shown that the selective Phospho1 inhibitor MLS-0263839 could protect VSMCs against calcification in chronic kidney disease models, confirming its pathological centrality in vascular mineralization (81). Within this mechanistic framework, recent studies have elucidated that nuclear receptor NR4A3 epigenetically activates Phospho1 through metabolic reprogramming. NR4A3 binds to the promoters of glycolytic enzymes, including aldolase A (ALDOA) and phosphofructokinase liver type (PFKL). This interaction enhances glycolytic flux and increases lactate production. The accrued lactate substrate drives histone lactylation, particularly at lysine 18 of histone H3, which epigenetically remodels the Phospho1 promoter into an open chromatin conformation. This lactylation-induced open chromatin state promotes recruitment of the transcriptional machinery and increases Phospho1 expression (82). NR4A3, functioning as an upstream metabolic orchestrator, directly binds to promoter regions of glycolytic genes ALDOA and PFKL, driving lactate overproduction through enhanced glycolytic flux (82). This lactate surge serves as the substrate for p300, a histone acetyltransferase that catalyzes the transfer of lactyl groups from lactyl-CoA to specific lysine residues on histones (6). By inducing lactate accumulation in VSMCs, NR4A3 promotes p300-mediated modification of histone H3 at lysine 18, a signature observed in calcified vascular tissues (82). Notably, p300 exhibits broader lactylation activity, targeting H3K14la and H3K9la in calcific aortic valve disease models, where these modifications exhibit site-specific regulatory roles: H3K14la enrichment at the bone morphogenetic protein 2 (BMP2) promoter facilitates its transcriptional activation, while H3K9la lactylation at RUNX2 enhancer regions stabilizes chromatin accessibility, synergistically amplifying osteogenic signaling (83, 84). This spatial specificity ensures coordinated upregulation of calcification drivers (BMP2, RUNX2) despite their distinct genomic loci. The pathological relevance of this axis is underscored by interventional studies: NR4A3 deficiency in chronic kidney disease models reduces p300-dependent H3K18la levels while attenuating aortic calcium deposition (82). Conversely, pharmacological p300 inhibition blocks lactylation-dependent BMP2 and RUNX2 expression without affecting intracellular lactate levels, confirming p300 as an executioner rather than metabolic regulator (83). Importantly, p300 activity depends on lactate concentration. Increased lactate production driven by NR4A3 further enhances p300-mediated lactylation. This feedforward loop promotes the transition of VSMCs toward a pro-calcific phenotype.

## 6 Lactylation in metabolic dysregulation associated with atherosclerosis

The development of AS is closely associated with metabolic dysregulation, particularly atherogenic dyslipidemia and insulin resistance. In addition to these well-established metabolic risk factors, emerging evidence indicates that lactylation also participates in the regulation of metabolic homeostasis through feedback mechanisms that fine-tune glycolytic flux and cellular metabolic balance.

### 6.1 Glycolysis-associated lactylation in metabolic feedback regulation

Enhanced glycolytic flux is a common metabolic feature under stress conditions and leads to increased lactate production, which provides a biochemical basis for lactylation modifications (17, 85). Glycolysis-derived lactate not only serves as a signaling molecule that drives inflammatory responses but may also participate in metabolic feedback regulation through lactylation. In this context, lactylation does not primarily amplify inflammatory signaling. Instead, it can restrain excessive glycolytic flux and promote metabolic adaptation. In particular, nucleolin (NCL), the most abundant nucleolar RNA-binding protein, is subject to lactylation at lysine 477 via p300 activity when glycolysis is hyperactive (86). This non-histone lactylation event suppresses glycolytic enzymes, including HK1, glucose-6-phosphate dehydrogenase (G6PD), and pyruvate kinase M (PKM). At the same time, it upregulates TCA cycle components. These changes limit excessive glycolysis and may attenuate plaque progression (87). Importantly, this suggests that NCL lactylation functions as a negative-feedback mechanism that limits metabolic overactivation rather than directly promoting inflammatory signaling. Similarly, site-specific lactylation of aldolase A (ALDOA) at lysine 147 (K147) directly inhibits its catalytic activity. Structural analysis suggests that this modification creates steric hindrance and impairs substrate binding. As a result, glycolytic flux is reduced. This process may establish a lactylation-mediated negative feedback loop in glycolysis (88). Together with NCL lactylation, this supports the view that lactylation can act as an intrinsic metabolic buffering mechanism under conditions of glycolytic stress. The functional consequences of lactylation are highly context-dependent. In contrast to its role in suppressing glycolysis within plaques, lactylation can promote pro-glycolytic gene expression in other settings, such as during Glis1-driven somatic cell reprogramming, where H3K18la facilitates the activation of pluripotency genes (89, 90). Taken together, these findings indicate that glycolysis-associated lactylation is not restricted to amplifying pathogenic signals but can also function as a homeostatic mechanism that restrains excessive glycolytic flux. Therefore, targeting specific lactylation sites, such as NCL K477 and ALDOA K147, may offer novel strategies to normalize dysregulated glucose metabolism in AS.

### 6.2 Lactylation in atherogenic dyslipidemia

As a prevalent metabolic risk factor, atherogenic dyslipidemia is characterized by elevated serum triglyceride and low-density lipoprotein (LDL) levels coupled with reduced high-density lipoprotein cholesterol levels (91, 92). Studies have suggested that peroxisome proliferator-activated receptor (PPAR) regulates the transcription of genes involved in lipid metabolism and energy homeostasis, and it may serve as a potential therapeutic target for the treatment of metabolic syndrome, particularly dyslipidemia (93, 94). Histone H3K18 (H3K18la) lactylation represents a key epigenetic mechanism linking cellular metabolic status to the transcriptional control of lipid metabolism. PPAR*γ* has been shown to mediate H3K18la deposition, which subsequently facilitates the recruitment of coactivators [steroid receptor coactivator 1/3 (SRC-1/3)] while concurrently displacing corepressors [nuclear receptor corepressor 1/2 (NCOR1/2)], thereby promoting the transcription of key genes involved in fatty acid oxidation (95). In addition, numerous studies have indicated that the transcriptional coactivator peroxisome proliferator-activated receptor gamma coactivator 1-alpha (PGC-1*α*) acts as an upstream regulator of lipid metabolism by enhancing the transcriptional function of PPAR*γ* (96). Notably, PGC-1*α* overexpression upregulates mitochondrial LDHB, shifting lactate metabolism toward pyruvate and consequently reducing lactate availability for protein lactylation. This decrease in mitochondrial protein lactylation, potentially affecting enzymes involved in *β*-oxidation such as acyl-CoA dehydrogenase medium chain (ACADM) and aldehyde dehydrogenase 7 family member A1 (ALDH7A1), stimulates fatty acid catabolism, ultimately leading to lower blood lipid content (97).

Lactate acts as an acyl donor and mediates the lactylation of apolipoprotein C-II (APOC2) at lysine 70 (K70), a reaction catalyzed by the histone acetyltransferase P300. APOC2 normally functions as a cofactor for lipoprotein lipase, promoting the hydrolysis of triglycerides into free fatty acids. Lactylation at K70 enhances the stability and lipolytic activity of APOC2, resulting in increased release of free fatty acids (FFAs). This dysregulation of lipid metabolism may subsequently promote lipid deposition within atherosclerotic plaques (98). Separately, histone lysine lactylation, a metabolic sensor associated with cellular plasticity, has been shown to participate in macrophage phenotypic switching during atherosclerosis. For instance, elevated histone lactylation in the late phase of M1 macrophage activation promotes Arg1 expression, facilitating wound healing and potentially mitigating persistent inflammation in atherosclerotic plaques (6). Mechanistically, fatty acid synthase undergoes lactylation at lysine 673 (K673), directly suppressing its enzymatic activity by sterically hindering the catalytic site His683, thereby reducing hepatic *de novo* lipogenesis. This regulatory mechanism is driven by mitochondrial pyruvate carrier 1 (MPC1)-dependent lactate accumulation, which acts as the precursor for lactylation (99). Pharmacologically, the herbal formula Huazhuo Tiaozhi granule (HTG) alleviates dyslipidemia by enhancing global protein lactylation, notably at histone H2B lysine 6 (H2BK6la) and histone H4 lysine 80 (H4K80la) in hepatocytes. Lactylome profiling revealed that HTG-induced lactylated proteins are significantly enriched in RNA processing pathways, including RNA-mediated gene silencing. Furthermore, histone H2B lactylation closely interacts with chromatin-modifying proteins such as chromodomain helicase DNA binding protein 4 (CHD4) and repressor element-1 silencing transcription factor (REST) corepressor 1 (RCOR1), suggesting that lactylation may facilitate chromatin remodeling. This epigenetic reprogramming likely underlies the downregulation of miR-155-5p, a microRNA known to promote hepatic lipid accumulation by impairing cholesterol efflux and disrupting lipid homeostasis. Consequently, HTG-mediated suppression of miR-155-5p attenuates hepatic lipid overload. This effect reduces serum total cholesterol and low-density lipoprotein cholesterol levels by enhancing hepatic lipid clearance and decreasing lipoprotein secretion (100). Collectively, lactylation regulates lipid metabolism and synthesis by modulating key metabolic enzymes and epigenetic reprogramming, providing a potential dual therapeutic strategy against atherogenic dyslipidemia and AS progression.

### 6.3 Lactylation in insulin resistance

Mounting evidence establishes a significant link between insulin resistance and AS pathogenesis (101, 102). Interestingly, emerging evidence indicates that lactate-induced lysine lactylation (Kla) serves as a novel regulatory mechanism in skeletal muscle insulin resistance. A recent study demonstrated that obese individuals exhibited elevated global protein lactylation levels in skeletal muscle, which were inversely correlated with insulin sensitivity. *In vitro* experiments confirmed that lactate directly induced global protein lactylation in a dose-dependent manner in primary human skeletal muscle cells. This increase in lactylation was accompanied by elevated phosphorylation of insulin receptor substrate-1 (IRS-1) at serine 636, a hallmark of impaired insulin signaling. This modification is associated with disrupted IRS-1 function in downstream PI3K/Akt pathway activation, thereby contributing to reduced glucose uptake. Furthermore, inhibition of lactate production via LDH-A siRNA or glycolysis blockade (2-DG) attenuated both global lactylation and IRS-1 phosphorylation, confirming lactate's role in this process. Notably, skeletal muscle lactylation levels were negatively associated with mitochondrial oxidative capacity *in vivo*. This association suggests that lactylation may contribute to insulin resistance by promoting anaerobic glycolysis and suppressing oxidative metabolism (103). Metformin, a well-established drug for diabetes treatment, directly reduces histone H3K18 lactylation by suppressing intracellular lactate accumulation. Experimental evidence from zebrafish and murine macrophage models demonstrated that metformin treatment significantly decreased lactate levels, accompanied by reduced H3K18 lactylation. Mechanistically, metformin inhibited glycolysis-related genes (PKM, glyceraldehyde-3-phosphate dehydrogenase) and lactate dehydrogenase (HDAC3), thereby limiting lactate availability for lactylation. Importantly, exogenous lactate supplementation restored H3K18 lactylation levels, confirming the direct dependence of lactylation on lactate metabolism. This suppression of H3K18 lactylation by metformin further attenuated ROS production and neutrophil recruitment (104). The evidence indicates that lactylation has significant potential in the regulation of insulin signaling and may represent a potential therapeutic target for limiting insulin resistance-related AS progression.

## 7 Conclusion

AS is a major driver of cardiovascular disease and has become one of the leading causes of mortality worldwide. Accumulating evidence suggests lactylation is involved in multiple pathological processes driving AS. This review summarizes the multifaceted roles of lactylation in the pathogenesis of atherosclerosis, highlighting its central role as a metabolic–epigenetic regulator across vascular inflammation, endothelial-to-mesenchymal transition, angiogenesis, vascular smooth muscle cell phenotypic switching, and metabolic dysregulation. First, lactylation exerts complex and context-dependent regulatory effects on vascular inflammation, demonstrating both pro-inflammatory and anti-inflammatory functions through distinct molecular mechanisms and cellular targets. Second, lactylation promotes EndMT by facilitating SNAI1 lactylation via MCT-dependent lactate uptake, thereby enhancing TGF-*β*/Smad2 signaling and inducing the expression of mesenchymal markers (e.g., *α*-SMA). In the context of angiogenesis, lactylation of HIF-1*α* stabilizes the protein under normoxic conditions, enabling transcriptional activation of CEMIP. This, in turn, promotes hyaluronic acid degradation and VEGFA-driven neovascularization, contributing to plaque destabilization. Regarding VSMC senescence and transdifferentiation, TRAP1-induced glycolytic reprogramming elevates H4K12la levels via suppression of HDAC3, thereby activating SASP genes. Concurrently, Sox10 lactylation, which depends on PI3K/AKT-mediated phosphorylation, directly upregulates macrophage-associated genes (e.g., C3, Cd74) to drive pro-inflammatory transdifferentiation of VSMCs. Third, lactylation contributes to metabolic dysregulation through multiple mechanisms. NCL lactylation at K477 suppresses glycolytic enzymes, including HK1 and PKM, while enhancing TCA cycle components such as succinate dehydrogenase complex subunit A and isocitrate dehydrogenase 3 gamma. These changes may counteract plaque hypoxia driven by the Warburg effect. In contrast, PPAR*γ*-mediated H3K18la inhibits fatty acid oxidation and exacerbates dyslipidemia. Furthermore, PGC-1*α* overexpression upregulates mitochondrial LDHB, shifts lactate metabolism toward pyruvate, and reduces substrate availability for mitochondrial protein lactylation, thereby promoting lipid homeostasis. Collectively, these findings indicate that lactylation serves as a key integrative mechanism linking metabolic reprogramming to epigenetic regulation in atherosclerosis.

However, our mechanistic understanding of lactylation's regulatory role in AS pathogenesis remains limited, and current evidence has several limitations that warrant further investigation. First, although lactylation promotes macrophage polarization toward an anti-inflammatory phenotype by upregulating Arg1 (6), it can also drive inflammation through PFKFB3-induced glycolytic flux, which increases H4K12la levels and activates NF-*κ*B (28). This paradox may arise from cell-type-specific lactylation dynamics or interactions with other post-translational modifications, such as acetylation, warranting further investigation into the spatiotemporal resolution of lactylation during different stages of inflammation. Second, lactylated proteins such as HMGB1 and HIF-1*α* participate in AS progression. However, their potential as diagnostic or prognostic biomarkers requires further investigation. Future research should validate lactylation profiles in clinical cohorts and develop non-invasive detection methods. Finally, the interplay between lactylation and other epigenetic modifications in AS, particularly acetylation, given the shared enzymatic regulators, such as p300/CBP, remains poorly understood and requires further investigation to elucidate potential synergistic or antagonistic crosstalk. Understanding these mechanisms may facilitate the development of novel therapeutic strategies targeting lactylation for the prevention and treatment of atherosclerosis and related cardiovascular diseases.

## Glossary

ACADM: acyl-CoA dehydrogenase medium chain
Akt/PKB: protein kinase B
ALDH7A1: aldehyde dehydrogenase 7 family member A1
ALDOA: aldolase A
ANGPT2: angiopoietin-2
APOC2: apolipoprotein C-II​
AS: atherosclerosis
ASF1A: antisilencing function 1A
Arg1: arginase 1​
Atg14L: autophagy-related protein 14-like protein
BCAP: b cell adaptor for phosphoinositide 3-kinase
BMP2: bone morphogenetic protein 2
CCL2: C-C motif ligand 2
CD31: cluster of differentiation 31
CEMIP: cell migration inducing hyaluronidase 1
CpG: cytosine–phosphate–guanine
ERK: extracellular regulated protein kinase
FOXO1: forkhead box O 1
EndMT: endothelial–mesenchymal transition
Glis1: Gli-similar 1
GSK3β: glycogen synthase kinase 3 beta
G6PD: glucose-6-phosphate dehydrogenase
HDAC2: histone deacetylase 2
HIF-1: hypoxia-inducible factor 1
HK: Hexokinase
HMGB1: high mobility group box 1
HSPA12A: heat shock protein A12A
HTG: huazhuo tiaozhi granule
ICAM-1: intercellular adhesion molecule-1
IKKβ: I (kappa) B kinase beta
IL: interleukin
IRS-1: insulin receptor substrate-1
LDH: lactate dehydrogenase
MAPK: mitogen-activated protein kinase​
MCTs: monocarboxylate transporters
MCP-1: monocyte chemoattractant protein 1
MECP2: methyl-CpG-binding protein 2
MECP2 K271la: MECP2 K271 lactylation
MI: myocardial infarction
MPC1: mitochondrial pyruvate carrier 1
NCL: nucleolin
NF-κB: nuclear factor-kappa B
NR4A: nuclear receptor subfamily 4, group A
mTOR: mechanistic target of rapamycin
ox-LDL: oxidized low-density lipoprotein
PAMPs: pathogen-associated molecular patterns
PFKFB3: 6-phosphofructo-2-kinase/fructose-2,6-bisphosphatase 3
PGC: peroxisome proliferator-activated receptor gamma coactivator
PI3K: phosphatidylinositol 3-kinase
PKM: pyruvate kinase M
ROS: reactive oxygen species
RAGE: receptor for advanced glycation end products
PFKL: phosphofructokinase, liver type
PGC-1α: peroxisome proliferator-activated receptor gamma coactivator 1-alpha
RGS5: regulator of G-protein signaling 5​
RhoA: RAS homolog family member A
RUNX: runt-related transcription factor
Pi: inorganic phosphate
PPAR: peroxisome proliferator-activated receptor
SASP: senescence-associated secretory phenotype
SNAI1: snail family transcriptional repressor 1
Sox10: SRY-box transcription factor 10​
TGF-β: transforming growth factor-β
Th17: T helper 17 cell
TLR: toll-like receptor
TRAP1: tumor necrosis factor receptor-associated protein 1
UVRAG: ultraviolet radiation resistance-associated gene
VCAM-1: vascular cell adhesion molecule 1
VE-cadherin: vascular endothelial cadherin
VEGF: vascular endothelial growth factor
Vps34: vacuolar protein sorting 34
VSMCs: vascular smooth muscle cells
